# Supplementary material for: The mechanism of eutectic growth in highly anisotropic materials
Source: Nat Commun. 2016 Sep 27;7:12953. doi: 10.1038/ncomms12953 (PMC5052661; doi:10.1038/ncomms12953)
Supplement: Supplementary Information — Supplementary Figures 1 - 4 and Supplementary References [file ncomms12953-s1.pdf]

Supplementary Figures

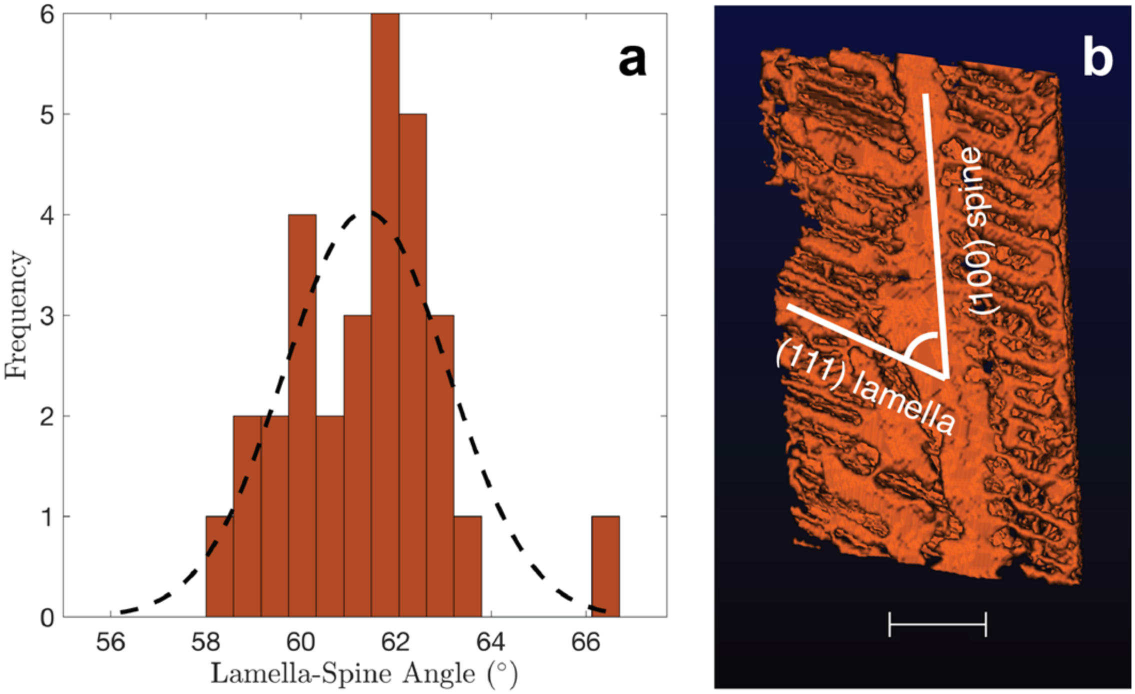

Supplementary Figure 1 (a) Distribution of lamella-spine angles, with mean angle of 61.4 ± 0.7°. This matches Hellawell's double-twin model.[1-3] (b) Back side of eutectic colony, showing Ge phase in orange. The lamellae-spine junction is indicated. Scalebar is 40 μm.

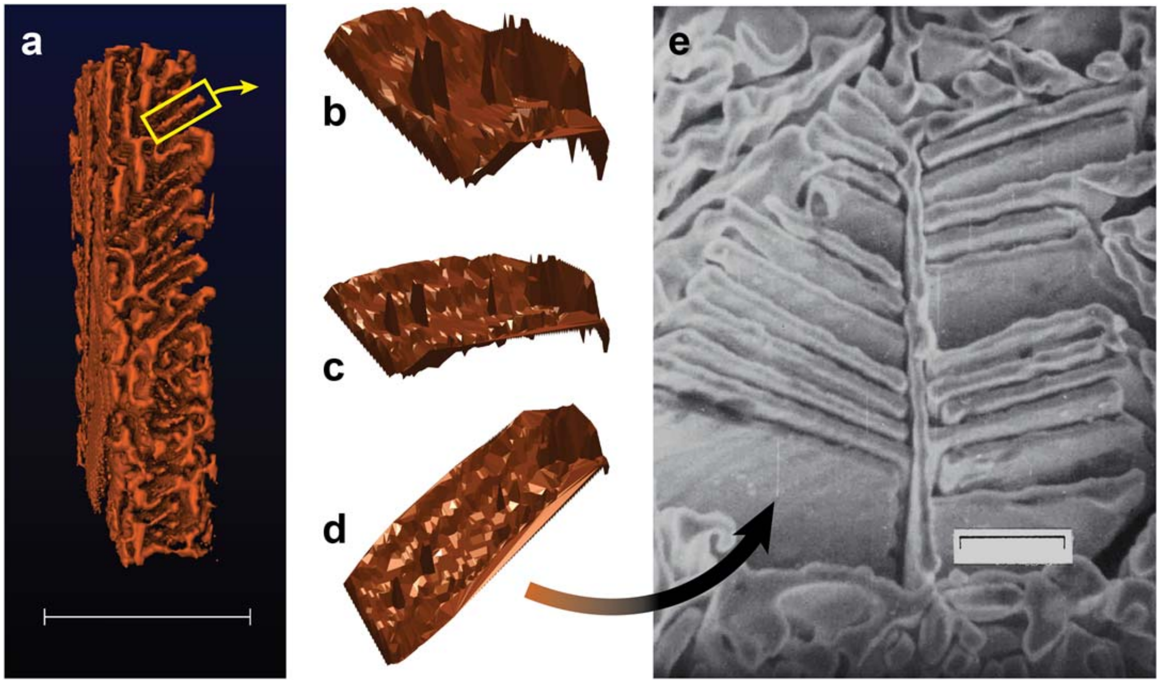

Supplementary Figure 2 (a) Side view of the eutectic colony, showing the Ge phase in orange. One of the regularly-arranged lamellae is boxed and isolated for further analysis. (b-d) Three views of the same lamella. Since the lamella is planar and runs the length of the spine, the (100) spine itself is likely defect-free. Any “roughness” in the plane of the lamella is due to small segmentation errors. (e) Comparison to the complex regular structure documented by Elliott.[4] Reproduced with permission from Elsevier.[4] Scalebars are (a) 80 μm and (e) 10 μm.

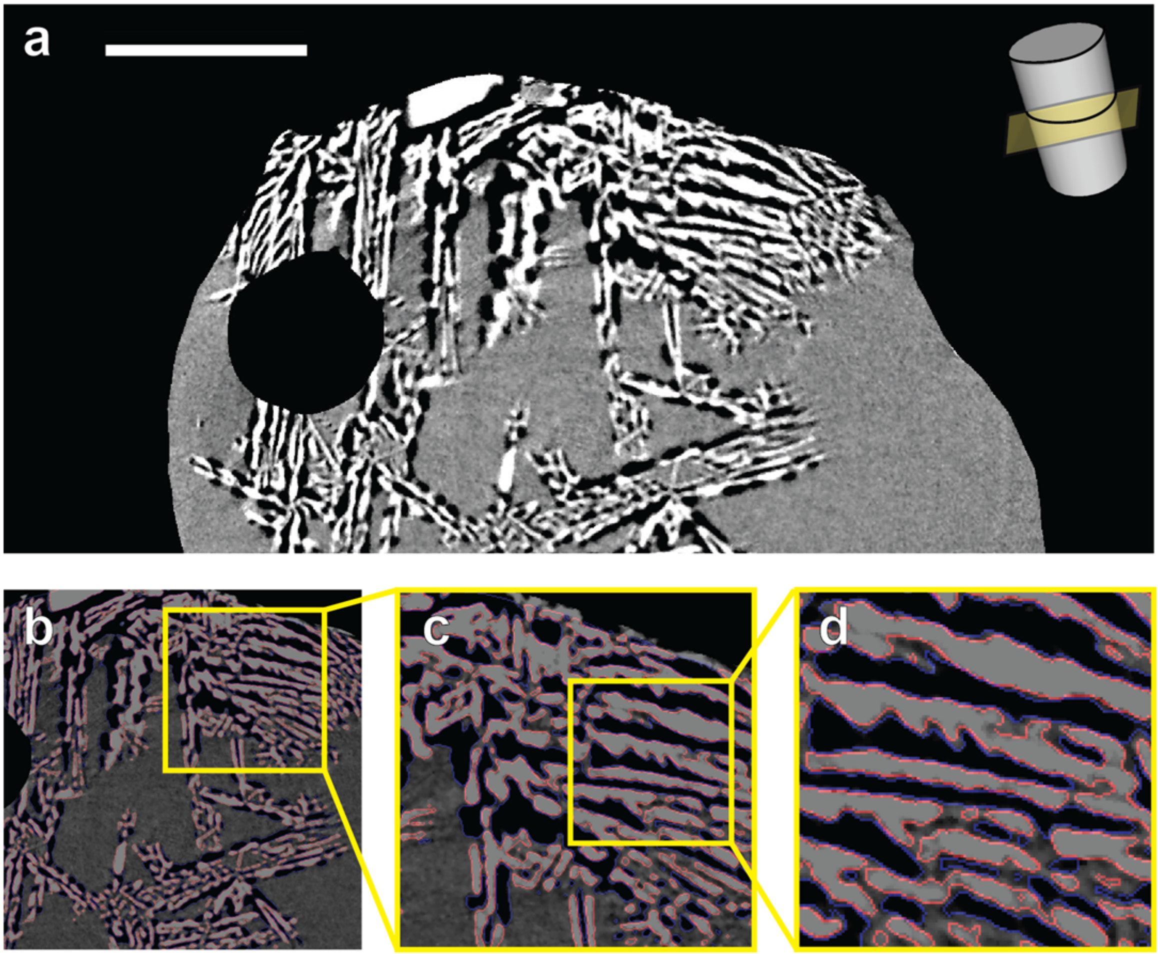

Supplementary Figure 3 (a) Representative 2D slice of 3D reconstruction, parallel to the axis of rotation of the cylinder-like sample (see inset). Ge is white, Al is black, and the liquid is gray. (b-d) Grayscale reconstruction across length scales, overlaid with edges of the segmentation output. Edges of Ge are orange and edges of Al are blue. The segmentation output shows excellent agreement with the eutectic structures underneath. Scalebar is 200 μm.

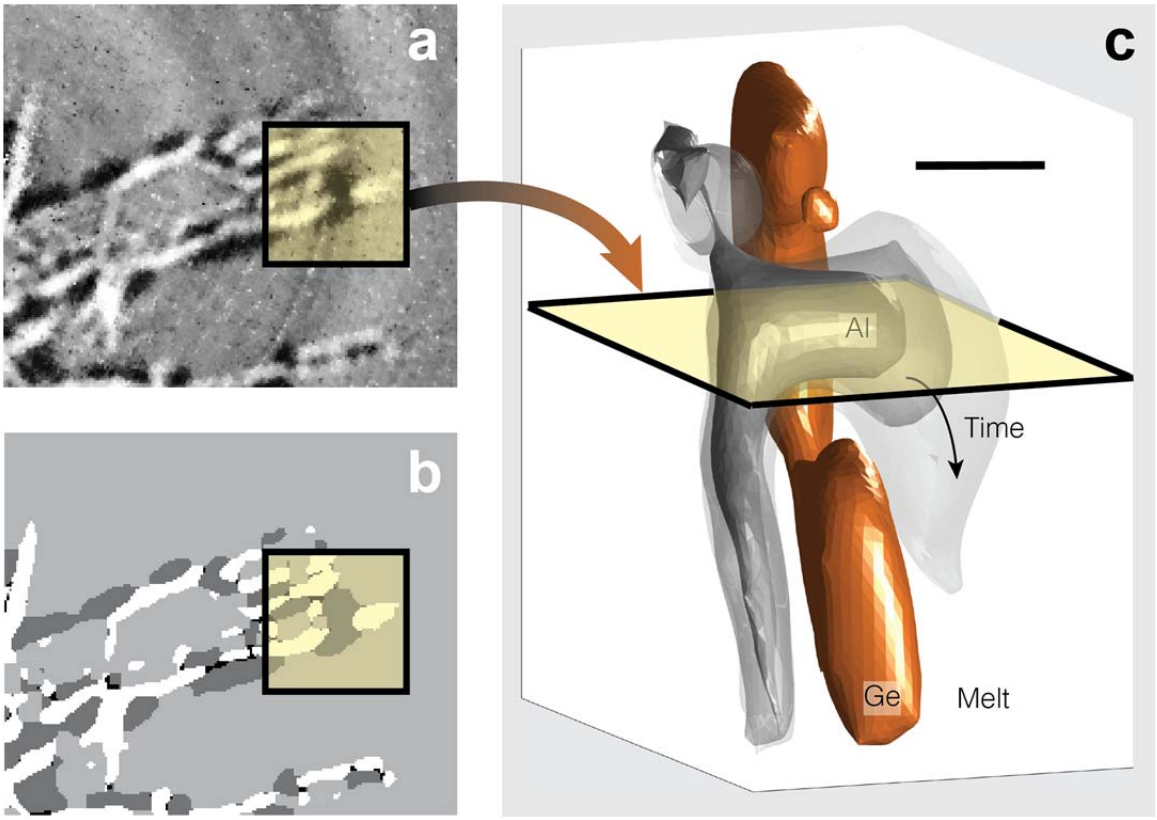

Supplementary Figure 4 (a) Grayscale region-of-interest (ROI) and (b) its segmentation. Ge is white, Al is dark gray, and the liquid is light gray. (c) 3D reconstruction (Fig. 2(e)) showing the same 2D ROI in the highlighted box. The 3D microstructure is consistent with the grayscale images: in both cases, Al emerges from holes or gaps in the Ge plates. Scalebar is 8 μm.

Supplementary References

1. Hellawell, A. The growth and structure of eutectics with silicon and germanium. *Prog. Mater. Sci.* **15**, 3–78 (1970).

2. Lu, S.-Z. & Hellawell, A. The mechanism of silicon modification in aluminum-silicon alloys: Impurity induced twinning. *Metall. Trans. A* **18**, 1721–1733 (1987).

3. Hellawell, A. The microstructure and crystallography of the aluminium-germanium eutectic. *Trans. Met. Soc. AIME* **239**, 1049–1055 (1967).

4. Elliott, R. *Eutectic Solidification Processing* (Butterworths, 1983).
